# Manufacturable 32-Channel Cochlear Electrode Array and Preliminary Assessment of Its Feasibility for Clinical Use

**DOI:** 10.3390/mi12070778

**Published:** 2021-06-30

**Authors:** Soowon Shin, Yoonhee Ha, Gwangjin Choi, Junewoo Hyun, Sangwoo Kim, Seung-Ha Oh, Kyou-Sik Min

**Affiliations:** 1 TODOC Co., Ltd., Seoul 08394, Korea; swshin@to-doc.com (S.S.); yhha@to-doc.com (Y.H.); gjchoi@to-doc.com (G.C.); hjw3152@to-doc.com (J.H.); tkddn8632@to-doc.com (S.K.); 2 Department of Otorhinolaryngology, Seoul National University Hospital, Seoul 03080, Korea; shaoh@snu.ac.kr

**Keywords:** cochlear implant, implantable neural interface, cochlear electrode array

## Abstract

(1) Background: In this study, we introduce a manufacturable 32-channel cochlear electrode array. In contrast to conventional cochlear electrode arrays manufactured by manual processes that consist of electrode-wire welding, the placement of each electrode, and silicone molding over wired structures, the proposed cochlear electrode array is manufactured by semi-automated laser micro-structuring and a mass-produced layer-by-layer silicone deposition scheme similar to the semiconductor fabrication process. (2) Methods: The proposed 32-channel electrode array has 32 electrode contacts with a length of 24 mm and 0.75 mm spacing between contacts. The width of the electrode array is 0.45 mm at its apex and 0.8 mm at its base, and it has a three-layered arrangement consisting of a 32-channel electrode layer and two 16-lead wire layers. To assess its feasibility, we conducted an electrochemical evaluation, stiffness measurements, and insertion force measurements. (3) Results: The electrochemical impedance and charge storage capacity are 3.11 ± 0.89 kOhm at 1 kHz and 5.09 mC/cm${}^{2}$, respectively. The V/H ratio, which indicates how large the vertical stiffness is compared to the horizontal stiffness, is 1.26. The insertion force is 17.4 mN at 8 mm from the round window, and the maximum extraction force is 61.4 mN. (4) Conclusions: The results of the preliminary feasibility assessment of the proposed 32-channel cochlear electrode array are presented. After further assessments are performed, a 32-channel cochlear implant system consisting of the proposed 32-channel electrode array, 32-channel neural stimulation and recording IC, titanium-based hermetic package, and sound processor with wireless power and signal transmission coil will be completed.

## 1. Introduction

A cochlear implant (CI) is a surgically implanted electronic medical device that restores sound perception for people with congenital or severe hearing loss by delivering modulated electrical stimulation to auditory nerves through an electrode array inserted in the cochlea. An intact cochlea has approximately 35,000–50,000 spiral ganglion cells, which sense the electrical potential induced by changes in the ionic concentration of synaptic connections with hair cells and generate the first auditory action potential. The deficiency or damage of cochlear hair cells causes sensory neural hearing loss (SNHL) because hair cells trigger the action potential in spiral ganglion cells. The location of the hair cells in the inner cochlea is related to the frequency that stimulates the corresponding spiral ganglion cells. The conventional multi-channel cochlear electrode array consists of 12–24 electrodes, lead wires and a silicone carrier. Each electrode stimulates ganglion cells at different locations, allowing the auditory nerve to deliver the corresponding sound frequency.

Since the first CI was introduced during the late 1970s, it has been considered the most successful treatment for SNHL. It is estimated that about 60,000–70,000 units of CI systems are implanted worldwide. Currently, the majority of CI units are distributed in developed countries because affordability remains a prohibitive challenge to people who need such devices in developing or under-developed countries. However, developing countries have started to estimate its cost-effectiveness in terms of the socioeconomic impact of hearing loss because the condition affects individuals in ways that limit their lives by depriving them of opportunities to be properly educated [1,2,3,4]. This includes impaired speech and language development and poor academic achievement, resulting in increased drop-out rates in adolescence. Therefore, CI has become a standard and cost-effective treatment for hearing loss, even in developing countries, where governments are beginning to provide free CIs to children [5,6,7]. Moreover, the social cost of hearing loss is being evaluated, as recent studies have reported that hearing impairment may accelerate the progress of dementia [8]. However, the expensive price of the device, ranging from USD 12,000 to 25,000, is still one of the greatest obstacles to obtaining CIs.

Efforts have been made to simplify devices and manufacturing processes to lower high CI prices. In 2007, a simplified CI was developed [9]. It had 16 ball-type electrodes to deliver eight-channel bipolar stimulation and used an off-the-shelf digital signal processor (DSP) to meet the minimum demands for effective sound perception, as articles have stated that more than eight channels of stimulation do not yield additional gains in speech recognition [10,11,12]. Researchers have focused on reducing costs by adopting semiconductor fabrication processes to reduce manual fabrication costs and to meet the minimum specifications [13,14,15]. They used liquid crystal polymer (LCP), a biocompatible, chemically inert, flexible, and thermoplastic material, as a substrate and insulation layer. The LCP-based cochlear electrode arrays include 16 gold contacts and lead wires patterned by photo-lithography and a chemical etching process. The most attractive aspect of the LCP-based cochlear electrode array is its thermoplastic bonding between the substrate and insulation layer. This property facilitates the production of a seamless and MR-compatible LCP-based implantable near-hermetic package as well as an electrode array [16,17,18]. These LCP-based CIs can replace current CI systems if they demonstrate their commercial and clinical feasibility by achieving ISO 10993 (International standards for biological evaluation of medical devices) certification and undergoing clinical studies.

The number of stimulation channels has been saturated for nearly 20 years. Increasing the number of stimulation channels has been considered unnecessary, as channel-to-channel electrical interference makes it difficult for CI users to distinguish the spatial resolution of the electrode array when adjacent electrodes are too close to each other. Though it has been asserted that more than eight channels are rarely effective in increasing the performance of speech perception, recent articles have reported results implying that the more channels that are activated, the higher the speech perception score achieved [19,20]. Researchers conducted speech perception experiments using a current 22-channel cochlear implant by activating 4, 8, 12, and 22 electrodes. Since conventional CI systems have 12–24 contacts of electrode and stimulation channels, further research using a greater number of channels could not be performed. To realize high-density CI electrode arrays with conventional methods, manufacturers should manually form more electrical contacts between electrodes and lead wires as many as the number of increased electrodes, but this will reduce production yield and further increase the cost. Therefore, innovations in conventional manufacturing methods using manual handling are needed to reduce CI costs and further advance clinical research using high-density CI electrode arrays with more than 24 electrical contacts.

In this article, we introduce a manufacturable 32-channel cochlear electrode array. In contrast to previous studies that utilized LCP as a substrate for the semiconductor fabrication process [14,15,16], we used conventional silastic materials for commercial feasibility. The electrode array consists of the inserted part, lead wires, and a pad array connected to feedthroughs. To simplify the interconnection process between electrodes and wires, 32-channel electrode and lead wire arrays are structured on a platinum–iridium film simultaneously using a pico-second laser, and then, a thin-film silicone 3D molding process is applied to form the silastic carrier. We assessed the feasibility through electrochemical and mechanical evaluation studies.

## 2. Materials and Methods

### 2.1. Design and Structure

**Figure 1:**
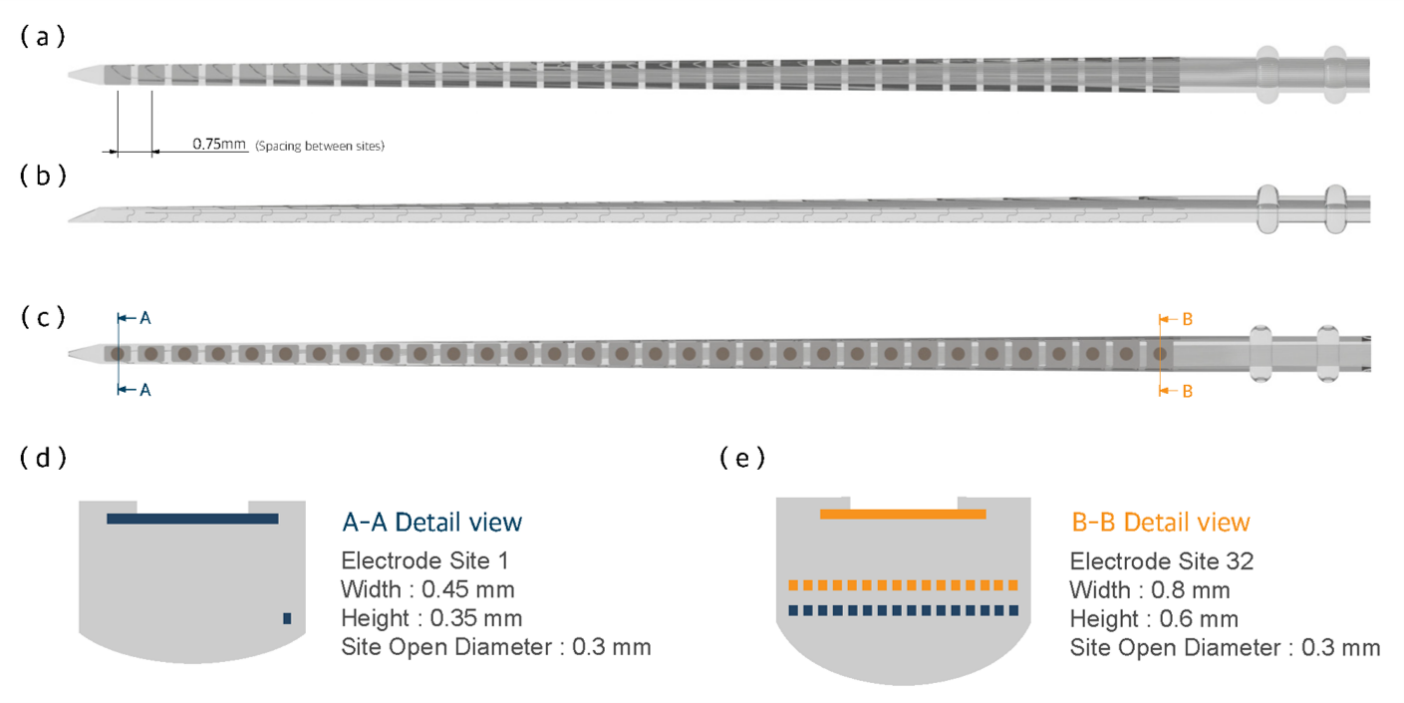
Design and structure of the proposed 32-channel electrode array. Images of (**a**) bottom; (**b**) side; and (**c**) top views of the electrode array. The cross-sectional images show the electrodes and the arrangement of the lead wires; (**d**) The cross-sectional view of the 1st electrode site from the apex. There is only one electrode contact and one lead wire inside the silastic carrier; and (**e**) the cross-sectional view of the 32nd electrode site from the apex. The 32nd electrode contact and 32 lead wires are inside the silastic carrier. The 32 lead wires are arranged in a 2×16 array.

Our 32-channel electrode array has 32 electrode contacts with a length of 24 mm and 0.75 mm spacing between contacts. The width of the electrode array is 0.45 mm at its apex and 0.8 mm at its base. These are comparable dimensions to conventional cochlear electrode arrays. Each electrode has a rectangular and planar shape because the electrode and lead wire arrays are structured by cutting platinum–iridium alloy foil. The smallest and the largest dimensions of the electrodes are 0.35×0.5×0.02 mm${}^{3}$ at the apex and 0.65×0.5×0.02 mm${}^{3}$ at the base, respectively. The site openings are circular holes on the silicone carrier that have 0.3 mm diameters. Figure 1 shows the bottom, side and top views of the electrode array. The cross-sectional view shows the electrodes and the arrangement of the lead wires. The lead wires are arranged in a 2×16 array instead of densely bundled wire arrays or an evenly distributed arrangement. The 16 wires on each plane are placed with 16 μm spacing.

### 2.2. Fabrication

The fabrication process of the proposed cochlear electrode array involves the following steps.

Automated laser micro-structuring: By applying laser micro-machining on 20 μm thick platinum–iridium alloy film (TANAKA HOLDINGS Co., Ltd., Tokyo, Japan), the 16-channel electrode and lead wire array module is structured using a pico-second laser (ProtoLaser R, LPKF, Garbsen, Germany) in a single process.

Mass-produced layer-by-layer silicone deposition: After laser micro-structuring, a thin-film silicone molding process is applied to the 16-channel electrode and wire array to form 20 μm-thick silastic insulation. Silicone by Nusil^®^ is applied by a dispenser (Ultimus I, Nordson EFD, Westlake, OH, USA) and cured by a 70 °C hot plate (HP330D, Misung Scientific, Seoul, Korea) for 30 min. Two modules consisting of 16-channel electrodes and wires are bonded using silicone to form the 32-channel electrode array. Each of the two 16-channel modules have alignment holes, which are used to align the two modules. For the bonding process, an alignment jig with alignment poles is used. After the bonding process, the silastic carrier is formed behind the electrode array with a pillar over the electrode site. Then, the electrode site is exposed by removing the pillar from the electrode array.

**Figure 2:**
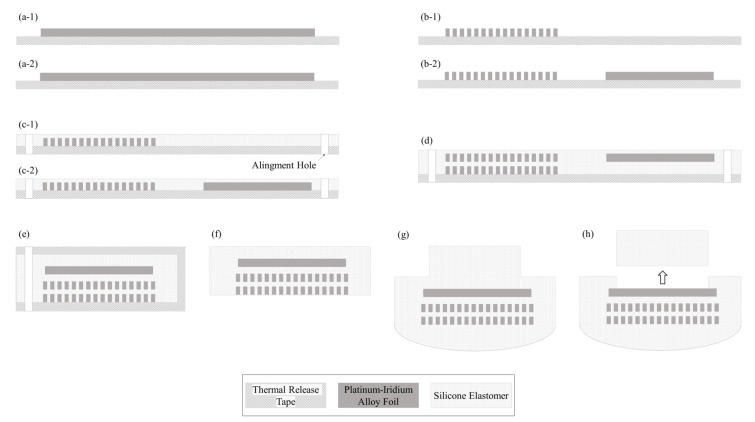
Fabrication Process: Cross-sectional view of electrode 32: (**a**) fixing two sheets of platinum–iridium alloy foil to each of the two thermal release tapes (Fine Technology Co., Ltd., Yangsan-si, Gyeongsangnam-do, Korea); (**b**) laser micro-structuring on platinum–iridium alloy foil; (**b-1**) laser micro-structured 1–16-channel electrode and wire module; (**b-2**) laser micro-structured 17–32-channel electrode and wire module; (**c**) applying thin-film silicone molding to two modules consisting of 16-channel electrode and wire arrays and forming alignment holes to the two modules; (**d**) bonding two modules of 16-channel electrode and wire arrays using silicone; (**e**) folding the 32-channel electrode and wire module to form a 3-layered arrangement: one 32-channel electrode layer and two 16-lead wire layers; (**f**) removing the sacrificial layer from the 32-channel electrode array; (**g**) forming silastic carrier behind the electrode array and applying the pillar over the electrode site; and (**h**) exposing the electrode site by removing the pillar.

Figure 2 shows the fabrication process for the 32-channel cochlear electrode array with a cross-sectional view of electrode 32.

### 2.3. Electrochemical Evaluation

To evaluate the characteristics of the manufactured 32-channel cochlear electrode array, electrochemical impedance spectroscopy (EIS) was performed on each electrode. The EIS of the electrodes was performed using a potentiostat (PalmSens4, PalmSens BV, Houten, The Netherlands) in a phosphate-buffered saline (PBS) solution (Gibco, Life Technologies, Paisley, UK) at pH 7.4 (1X) with a three-electrode system. An Ag/AgCl electrode and Pt wire were, respectively, used as reference and counter electrodes. The electrochemical impedance was measured in the frequency range of 100 Hz–10 kHz.

In addition, cyclic voltammetry (CV) was used to calculate cathodic charge storage capacitance (CSCc) for the analysis of electrode properties. The CV curve was measured after 100 cycles with a scan rate of 0.1 V/s within a voltage range of −0.6 V to 0.8 V versus the Ag/AgCl reference electrode. The area within the curve was calculated using PSTrace (PalmSens BV, Houten, The Netherlands) software to obtain the CSCc.

### 2.4. Stiffness Measurement

**Figure 3:**
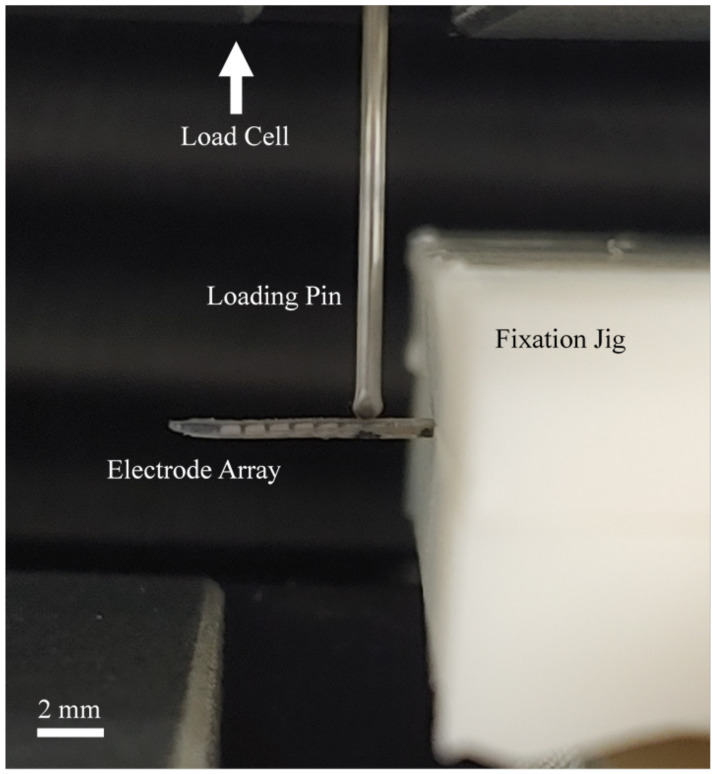
Deflection force measurement. The electrode arrays were fixed to a custom fixation jig, and a loading pin attached to a load cell was used to bend the electrode array 30° at a distance of 2 mm from the fixed point.

The stiffness of an intracochlear electrode array can affect the insertion results, such as the occurrence of insertion trauma and the insertion depth within the cochlea. Therefore, the stiffness of the proposed electrode array was measured (Figure 3). The fabricated electrode arrays were fixed to a custom fixation jig, which was attached to a universal testing machine (UTM, QM100S, QMESYS Co. Ltd., Uiwang-si, Gyeonggi-do, Korea). A load cell in the UTM was used to measure the deflection force required to flex the electrode array 30° from its normal shape. Stiffness in the vertical and horizontal planes was measured at 6 mm from the apex and 2 mm from the fixed point of the fixation jig. Each measurement was performed in both directions, and the values measured with three electrode arrays were averaged. Additionally, the stiffness ratio (V/H ratio) was calculated as the ratio of vertical stiffness to horizontal stiffness.

### 2.5. Insertion and Extraction Force Measurement

Insertion and extraction tests were performed to assess the insertion depth and force exerted by the fabricated electrode array. The electrode array and a plastic human ST (Scala Tympani) model were fixed to the upper and lower grips of the UTM, respectively. Electrode arrays were gradually inserted in and extracted from the ST model at a speed of 0.6 mm/s. The ST model was filled with phosphate-buffered saline solution as a lubricant to mimic the human cochlear environment [21]. During electrode insertion and extraction, a load cell in the UTM recorded the displacement and the instantaneously exerted forces. The electrode array was tested 10 times, and the average insertion and extraction forces are reported in this study.

## 3. Results

### 3.1. Thirty-Two-Channel Cochlear Electrode Array

**Figure 4:**
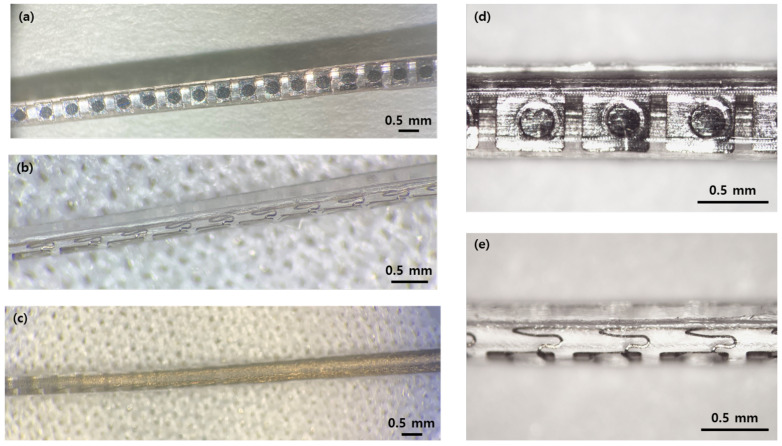
Manufactured 32-channel cochlear electrode array. Pictures of: (**a**) top; (**b**) side; and (**c**) bottom views of the electrode array. Enlarged pictures of (**d**) top and (**e**) side views.

The manufactured 32-channel cochlear electrode array is shown in Figure 4. As shown in the top view, the electrode sites are exposed as circular holes with 0.3 mm diameters. The side view shows that the silastic carrier is formed behind the electrode and wire bundle. Since the fabricated 32-channel electrode array has a three-layered arrangement consisting of a 32-channel electrode layer and two 16-lead wire layers, where only the wire bundle is shown in the bottom view of the electrode.

### 3.2. Electrochemical Evaluation

**Figure 5:**
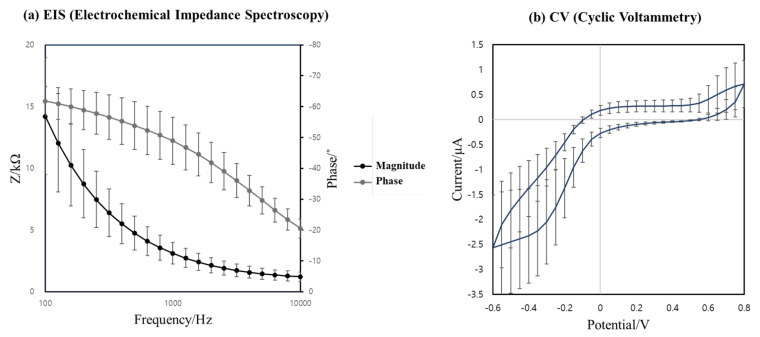
Electrochemical properties of the manufactured 32-channel cochlear electrode array: (**a**) Electrochemical impedance spectroscopy in a frequency range of 100 Hz–10 kHz: average impedance magnitude (black) and average phase angle (gray); and (**b**) cyclic voltammetry of the electrodes was measured within a voltage range of −0.6 V to 0.8 V versus the Ag/AgCl reference electrode.

To evaluate the electrochemical properties of the manufactured 32-channel cochlear electrode array, EIS and CV were measured, and CSCc values were calculated from the measured CV curves, as explained in the methods section. The average electrochemical impedance magnitude and phase angle of the 32 electrodes are 3.11 ± 0.89 kOhm and −48.9 ± 7.61° at 1 kHz, and the average CSCc is 5.09 mC/cm${}^{2}$, as shown in Figure 5.

### 3.3. Stiffness Measurement

To quantify the deflection force exerted by the fabricated electrode array, the stiffness was measured by flexing the array. In addition, the stiffness of the electrode array was compared to that of a conventional cochlear electrode array manufactured by Nurobiosys (Seoul, Korea) (Table 1). The Nurobiosys electrode array is a 16-electrode array made with 16 ball contacts and wires in a silicone carrier [9,22]. The vertical stiffness of the proposed electrode array is 19.8 mN, and the horizontal stiffness is 15.7 mN, with an average of 17.8 mN. The calculated stiffness ratio (V/H ratio) is 1.26, which shows that the electrode array has greater stiffness in the horizontal plane. Both the vertical and horizontal stiffness of the fabricated electrode array are less than those of the conventional electrode array.

It has been reported that the occurrence of electrode insertion trauma is more related to the V/H stiffness ratio than the overall stiffness [22]. Electrode arrays with greater stiffness in the vertical plane are less likely to induce insertion trauma. The electrode array presented in this paper has greater vertical stiffness than horizontal stiffness (V/H ratio of 1.26), which might have the advantage of facilitating atraumatic insertion. The effect of the V/H ratio can be confirmed through the results of a human temporal bone study that shows no observable trauma.

The V/H ratio of the electrode array may be the result of the fabrication method based on platinum–iridium films. Because the electrode wires patterned by the pico-second laser are aligned on the vertical plane, the electrode array is expected to have greater stiffness in the vertical plane.

### 3.4. Insertion and Extraction Force Measurement

**Figure 6:**
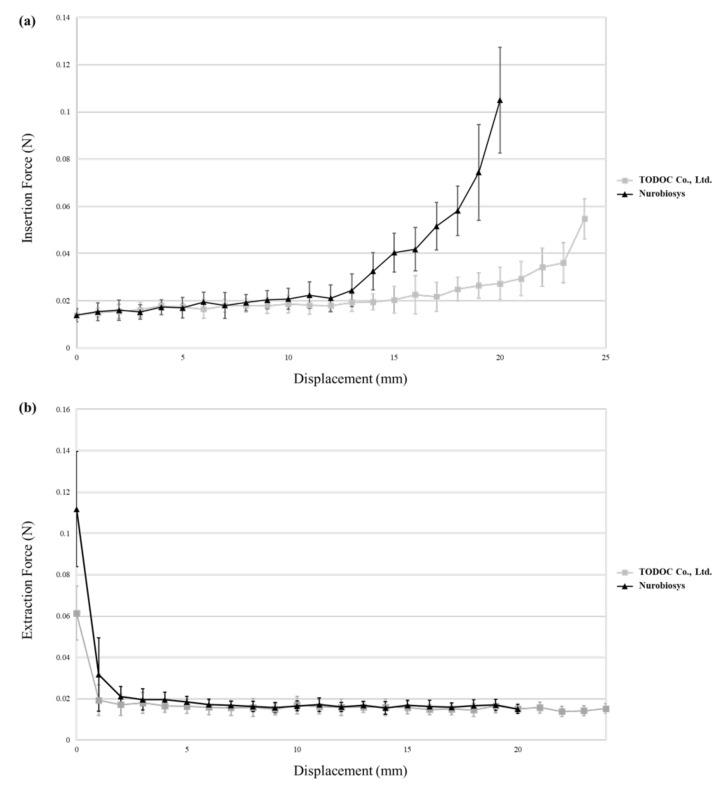
(**a**) Insertion and (**b**) extraction force measurements. Average and standard deviation of measured values are shown.

The insertion and extraction forces of the fabricated electrode array were measured and compared to those of Nurobiosys’s metal wire-based electrode array, as shown in Figure 6. All active stimulation ranges (24 mm) were applied to the human ST model throughout the entire insertion process. The insertion force of the fabricated electrode array is 17.4 mN at 8 mm from the round window, and the maximum extraction force is 61.4 mN. For the metal wire-based electrode array manufactured by Nurobiosys, the insertion force at a displacement of 8 mm is 19.2 mN, and the maximum extraction force is 111.8 mN, with an active stimulation range of 20 mm. Both the insertion and extraction forces of the fabricated electrode array are less than those of the conventional electrode array when the arrays are placed near the final position in the ST model.

## 4. Discussion

### 4.1. Cost and Feasibility

The implantable pulse generator (IPG) of the conventional CI consists of an intracochlear electrode array, lead wires, electronics, a hermetic package and a coil for wireless telemetry [23]. Usually, platinum or platinum–iridium alloy is used for the intracochlear electrode array and lead wires because platinum-based material meets the requirements of biocompatibility, long-term reliability, and charge storage capacity for neural stimulation. However, according to the annual report of the leading company, the high device costs do not appear to be material costs, as manufacturing costs and operating expenses account for 24% and 50% of their expenditure, respectively. In developing countries, thousands of CI units are supplied by tender at about USD 6000 per unit. In 2012, the number of employees at Cochlear Limited was 2390, and the company supplied 23,087 CI units [24]. More recently, in 2019, about 4000 employees were working for the company, and 34,083 CI units were provided globally [25]. To manufacture more devices, additional facilities and employees are needed, which will increase the fixed financial burden. While device prices have been cited as the greatest prohibitive barrier in the provision of CIs in recent decades, in fact, a more fundamental cause might be productivity. Among the components of the implantable pulse generator, a fine cochlear electrode array as small as 0.4–0.8 mm in diameter and 20–25 mm in length is manufactured by manual fabrication, which includes forming the electrode, welding electrodes and lead wires, positioning each electrode and wire on the mold, and silicone injection molding.

In 1983, Clark et al. stated that a multi-channel cochlear implant should meet the following requirements: (1) atraumatic insertion and extraction; (2) biocompatibility; (3) localization to discrete groups of nerve fibers; (4) long-term chemical reliability; (5) mechanical robustness and stability; and (6) simple and inexpensive fabrication process [26]. Based on their work, the current multi-channel CI electrode array employs platinum alloy-based electrodes and wires and a silastic carrier. Platinum and platinum–iridium alloys are common materials used for the electrical stimulation of excitable tissue [27]. The thickness of the electrode contact should be sufficient to sustain long-term stimulation because the stimulation is accompanied by corrosion [28]. Commercial cochlear implants employ platinum-based electrode contacts thicker than 10 μm [29].

Micro-machined CI electrode arrays have been introduced in studies employing micro-LEDs, drug-eluting scaffolds, thin-film fabrication, etc. [30,31,32,33]. However, the integration of lead wires should be considered in order to deliver these excellent technologies to people with hearing loss. The lead wire array, spanning from the pulse generator placed behind the ear to the round window, should be as long as 70–100 mm. ISO 14708-7 (International standard for particular requirements for cochlear and auditory brainstem implant systems) states that implantable leads outside the stimulator shall withstand the tensile forces that might occur during or after implantation, without fracture of any conductor or deterioration to any functional electrical insulation [34]. In addition, the standard requires CI devices to withstand harsh tests, such as multiple-drop, flexibility, or elongation tests. Given these requirements, the length of the micro-machined CI electrode array should not be merely 20 mm but at least 120 mm if it includes lead wire arrays. Moreover, connectivity between the lead and feedthrough arrays should be applied to the design. For this reason, not many electrode arrays can be patterned on the 6–8-inch wafers used in the semiconductor fabrication process, so it is not expected to significantly reduce CI costs from the current price.

The device that we introduce in this article has two implications: (1) the suggestion of a compromise between mass production using a MEMS process and conventional manual fabrication; (2) the development of a feasible platform for clinical research using a high-density CI electrode array. The MEMS process is ideal for micro-devices when thousands of units can be fabricated on a single process-compatible wafer. This means that many micro-devices are distributed on the surface of the wafer, which can be used in most cases, even if a small number of defects occur during the process. However, for a CI electrode array over 100 mm in length, only dozens of products can be fabricated on a wafer [35], so small defects in the pattern can significantly reduce the yield of the process. In addition, considering the cost of facility investment and maintenance in the semiconductor process, the cost of the facility could make the product more expensive, given that the current sales volume is only tens of thousands of CI units per year [36]. CI electrodes developed using a MEMS process are quite suitable for high-performance CI with high-density electrodes that cannot be fabricated manually. The laser micro-machining process suggested in this work offers an appropriate compromise between inexpensive production and a high-performance CI electrode array because it patterns the microstructure on platinum–iridium alloy film, forming 32-channel electrode, lead, and pad arrays without manual handling. Conventional MEMS-based high-density CI electrodes employ semiconductor process-compatible polymeric substrates [13,14,15,31]. However, because most of these materials and fabrication processes have not been used in chronic active medical implants, entire implant systems should undergo biocompatibility tests based on ISO 10993 to proceed to clinical studies [37]. Thus, it is difficult to apply these high-density electrode arrays fabricated on brand new material to clinical studies unless medical device manufacturers are willing to take responsibility for unintended biocompatibility issues. This work offers a clinical research platform using high-density CI electrode arrays because none of the materials used for the electrode array differ from conventional materials compliant with ISO 10993 [23]. The authors and colleagues also designed a 32-channel receiver–stimulator IC, a titanium-based hermetic package, transceiver coils, and a 32-channel sound processor in preparation for applying the electrode array to a full CI system.

### 4.2. Design Challenges for High-Density CI Electrode Array

Conventional MEMS CI electrode arrays are patterned on two-dimensional surfaces, which means that electrodes and wires are on the same plane. Because CI electrode arrays require narrow structures, as small as 0.3–0.4 mm at the tip and 0.6–0.8 mm at the base, it is challenging to establish a sufficient area for the electrode to deliver effective electrical stimulation. For example, in a previous work based on LCP [14], the width and the spacing of basal wires were 10 μm. Thus, the lead wires of 16 electrodes occupied a 320 μm wide area at the base of the electrode array. Therefore, in a successive work, the wire patterns were placed on the sub-plane to reduce the width of the electrode array [38]. However, as described above, the LCP-based electrode array uses gold electroplating to thicken the pattern on each layer of the electrode and wire array. However, it is difficult to integrate thick platinum layer by layer in the same way. For this reason, we chose to pattern the electrodes and wires in the same plane using one thick platinum layer. In this work, we only placed lead array patterns on one side and electrodes on the other, relative to the folding line, in order to form a three-dimensional multi-layered structure after folding. Because 16 μm-wide wires with the spacing needed for 32 channels require a width exceeding 1 mm in the basal area, we adopted a modular fabrication approach with two 16-channel electrode arrays so that the widest area required for the lead array does not exceed 0.7 mm.

**Figure 7:**
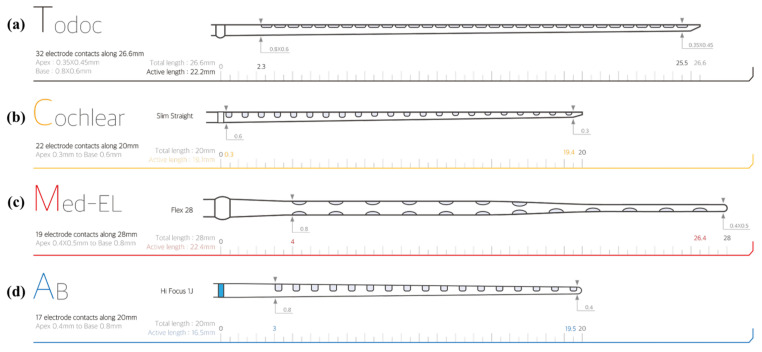
Comparison of Commercial CI Electrode Arrays: (**a**) TODOC Co., Ltd.; (**b**) Cochlear Ltd.; (**c**) Med-El; and (**d**) Advanced Bionics.

The longest commercially available CI electrode array is a Med-El FLEXSOFT^TM^, which has carrier and active lengths of 31.5 mm and 26.4 mm, respectively [29]. The spacing between electrodes is 2.4 mm because they have 12 stimulation channels. This kind of full-length CI electrode can be useful for completely deaf CI users because the longer the electrode array, the deeper the cochlear region that can be stimulated. On the other hand, Slim Straight, which has a 25 mm carrier length, is among the longest electrode arrays manufactured by Cochlear Limited. As shown in Figure 7, Slim Straight has the smallest diameter among electrodes. This property is helpful for preserving residual hearing after surgery [39]. Based on the goal of realizing a manufacturable process, the authors of this study designed the dimensions of the first electrode array to be between those of FLEXSOFT^TM^ and Slim Straight. The spacing of the proposed electrode array is 0.75 mm. Because the length of the proposed electrode array is only 24 mm, the spacing between electrodes is not as wide as that in commercial CI electrode arrays. However, the electrode array that has the highest density in terms of spacing between electrodes is Slim Modiolar by Cochlear Limited, as it has 22 contacts along a 14 mm span of active length. Because the presented electrode array is the first full-length high-density electrode array with more than 30 contacts, further clinical studies, such as electrode selection for patients with neural dead regions and changes in the performance of full-channel stimulation using more than 22 channels, will be possible with it.

### 4.3. Mechanical Property

Several factors determine the mechanical properties of the CI electrode array [29]. This section describes one of them: the thickness of each lead wire and the cross-sectional arrangement of the lead wire array. The material and dimensions of the lead wire were determined by considering the electrical conductivity, stiffness and the ease of manufacturing the wire. Because the number of lead wires increases from the apex to the base, the stiffness at the apex is far less than that at the base. Appropriate stiffness of the electrode array is essential for inserting the electrode deeper and minimizing the insertion trauma. To realize atraumatic insertion, commercial CI electrode arrays employ teflon-coated platinum–iridium fine wires with diameters of 0.025–0.03 mm. The larger the diameter and the greater the number of lead wires, the stiffer the electrode array becomes. If the 32-channel CI electrode array employs commercial fine wires, the basal part of the electrode becomes too stiff to be inserted fully in the cochlea. As shown in Figure 1, the cross-sectional view of the electrode array in this study contains rectangular wire arrays. Each wire has dimensions of 16 × 20 μm. The dimensions of the cross-section of the wires can be adjusted if the device is too stiff or too soft to be inserted in the cochlea. The wire array is designed to be as narrow as possible at the inserted part to make it softer and wider at the lead part to increase the conductivity of each channel.

**Figure 8:**
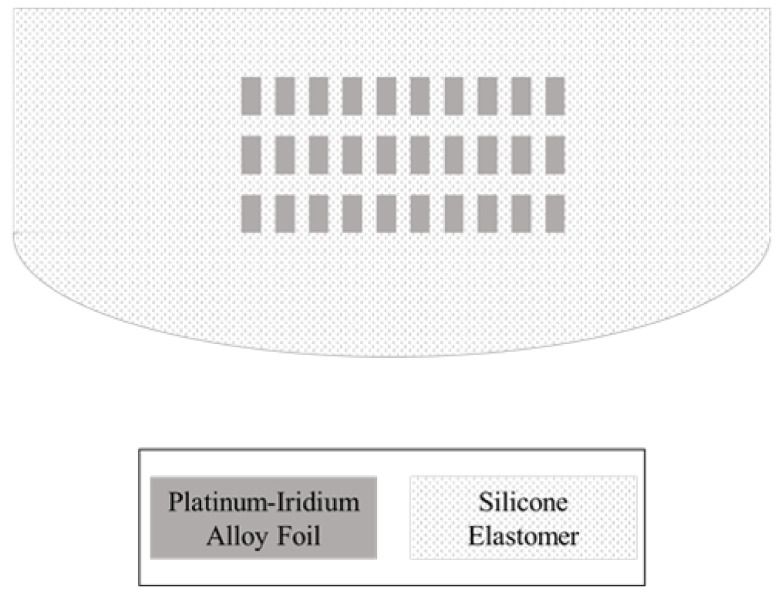
Structure of the triple-layer wire bundle. Thirty wires are placed in 3 layers, each with 10 wires.

**Figure 9:**
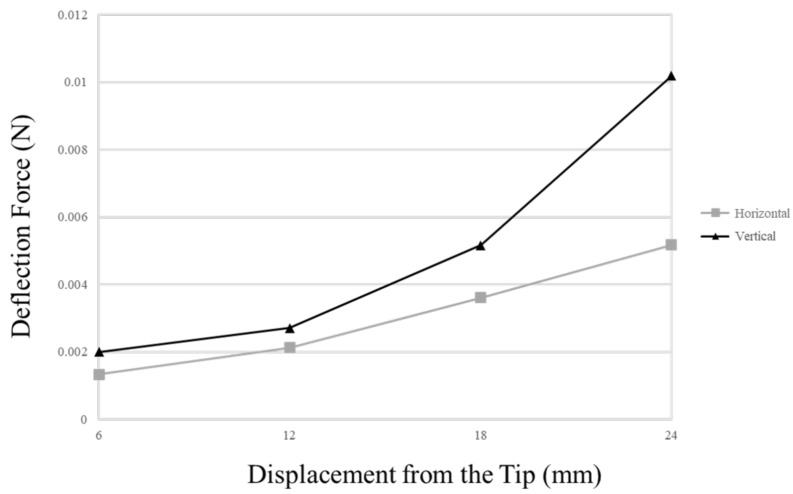
Deflection force measurements of the triple-layer wire bundle in both the horizontal and vertical planes. Deflection forces required to bend the electrode array 30° from its normal shape were measured at 6 mm increments from the tip to the base.

If electrodes are manually manufactured, the cross-sectional arrangement of lead wires is difficult to control. Cross-sectional wire arrangement can affect the insertion behavior because the deflection force can vary according to the distance among wires. Therefore, most current CI manufacturers form straight wire bundles that are as dense as possible to reduce the deflection force of the electrode array. In contrast to other CI manufacturers, MED-EL electrodes uniformly distribute the wavy wires in the silicone carrier, which helps distribute the forces and prevent the electrode from behaving like a needle and causing damage during insertion [29]. To compare the deflection force of the wire bundle according to the direction in which the wire bundle array is formed, we manufactured a 30-wire bundle with a total of three layers, as shown in Figure 8, each consisting of 10 wires, by the method proposed in Section 2.2. The deflection force required to bend the electrode array 30° from its normal shape in both horizontal and vertical directions was measured at 6 mm increments from the tip to the base. Figure 9 shows the deflection force measurement results of the manufactured wire bundle. In both directions, the number of lead wires proportionally increases as the measurement point moves from tip to base. The measured horizontal deflection force is proportional to the increase in the number of wires in the horizontal direction, i.e., the number of layers in the wire bundle. In the vertical direction, on the other hand, the deflection force is measured relative to the number of wires in the vertical direction, that is, the number of wires that make up a single layer. In other words, Figure 9 shows the results of measuring the deflection force by increasing the number of wires for a bundle of 10×1 to 10×3 wires in the horizontal direction and a bundle of 3×1 to 3×10 wires in the vertical direction. As the deflection force proportional to the increase in the number of wires has been shown to increase more rapidly in the vertical direction than in the horizontal direction, it is desirable to place the additional wires in the horizontal direction as much as possible. Because of these properties, the electrodes proposed in this paper exert less insertion force while involving at least 10 more electrodes and lead wires compared to the metal wire-based electrodes by Nurobiosys (Figure 6).

### 4.4. Future Research Direction

**Figure 10:**
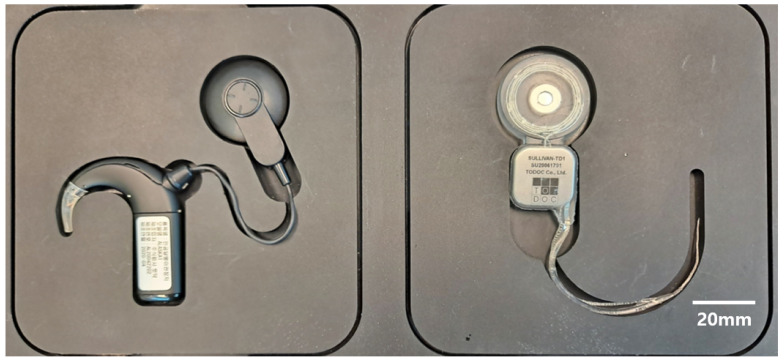
TODOC’s 32-Channel Cochlear Implant Sound Processor (**Left**) and Implantable Pulse Generator (**Right**). The 32-channel cochlear implant system consists of 32-channel neural stimulation and recording IC, titanium-based hermetic package, and sound processor with wireless power and signal transmission coil.

This article describes the fabrication process and preliminary feasibility assessment of a 32-channel CI electrode array. For further assessment, a human temporal bone insertion study is being conducted. After a preliminary cadaver safety study, a comparative human temporal bone study with a larger sample size is needed that compares the device to other commercial electrodes. The cochlear implant is one of the most complicated implantable medical devices. In order to deliver the results of this study to hearing-impaired people, further work on the implantable hermetic package, neural stimulation and recording circuit, wireless power and signal telemetry, and sound processing should be completed. The authors and colleagues are currently developing a 32-channel cochlear implant system consisting of 32-channel neural stimulation and recording IC, a titanium-based hermetic package, and a sound processor with wireless power and a signal transmission coil (Figure 10).

## 5. Conclusions

In this article, the fabrication process and electrochemical and mechanical properties of a 32-channel CI electrode array were introduced. The proposed 32-channel electrode and lead wire array was manufactured using automated laser micro-machining and a three-dimensional micro-molding process. The average impedance magnitude of the 32 electrodes is 3.11 ± 0.89 kOhm at 1 kHz. The average CSCc is 5.09 mC/cm${}^{2}$. The insertion force of the fabricated electrode array is 17.4 mN at 8 mm from the round window, and the maximum extraction force is 61.4 mN, which is comparable to the conventional CI electrode.

To complete the 32-channel cochlear implant system with the electrodes proposed in this paper, the authors and colleagues are working on the development of other parts that are needed to make up the system. In the near future, we expect to introduce our 32-channel cochlear implant system, consisting of 32-channel neural stimulation and recording IC, titanium-based recluse packages, wireless power, and sound processors with signal transmission coils.

## Figures and Tables

**Table 1:** Electrode stiffness measurement results.

Name	Vertical	Horizontal	Mean	
of	Stiffness	Stiffness	Stiffness	V/H Ratio
Manufacturer	(mN)	(mN)	(mN)	
**TODOC Co., Ltd.**	**19.8**	**15.7**	**17.8**	**1.26**
Nurobiosys	25.2	21.4	23.3	1.18
